# Preclinical evaluation of ribociclib and its synergistic effect in combination with alpelisib in non-keratinizing nasopharyngeal carcinoma

**DOI:** 10.1038/s41598-018-26201-1

**Published:** 2018-05-22

**Authors:** Chi-Hang Wong, Brigette B. Y. Ma, Connie W. C. Hui, Kwok-Wai Lo, Edwin P. Hui, Anthony T. C. Chan

**Affiliations:** 1 Cancer Drug Testing Unit (CDTU), Hong Kong Cancer Institute and Li Ka Shing Institute of Health Sciences, The Chinese University of Hong Kong, Hong Kong SAR, People’s Republic of China; 2 Department of Clinical Oncology, State Key Laboratory in Oncology in South China, Sir YK Pao Centre for Cancer, The Chinese University of Hong Kong, Hong Kong SAR, People’s Republic of China; 3 Department of Anatomical and Cellular Pathology, The Chinese University of Hong Kong, Hong Kong SAR, People’s Republic of China

## Abstract

Ribociclib is a specific cyclin dependent kinase (Cdk) 4/6 inhibitor that induces G_1_ arrest by blocking the formation of cyclin D1-Cdk4/6 complex and inhibiting retinoblastoma (RB) phosphorylation. Cyclin D1 is overexpressed in over 90% of nasopharyngeal carcinoma (NPC) and CCND1 gene activation plays a critical role in NPC pathogenesis. This study evaluated the preclinical activities of ribociclib in NPC cell lines and patient derived xenograft (PDX) models. Over 95% cell growth inhibition was observed at 96 hours after ribociclib treatment. (IC_50_ concentrations: HK1 = 1.42 ± 0.23 µM; HK1-LMP1 = 2.18 ± 0.70 µM and C666-1 = 8.26 ± 0.92 µM). HK1 and C666-1 cells were chosen for analysis of ribociclib on kinase signaling, apoptosis and cell cycle. Treatment with ribociclib for 48 hours consistently showed a dose-dependent reduction in phosphorylated and total RB expression and G_1_ cycle arrest was only observed. Combining ribociclib with the alpha-specific PI3K inhibitor alpelisib showed a synergistic effect in two NPC PDX models in nude mice. The co-treatment induced a significant reduction in tumor volume in both xeno-666 and xeno-2117 compared with ribociclib treatment alone and control (p < 0.01). In summary, ribociclib is active in NPC models and the effect on growth inhibition was augmented when combined with alpelisib. This study supports the clinical evaluation of ribociclib in NPC.

## Introduction

Non-keratinizing nasopharyngeal carcinoma (NPC) is endemic to Southern China and certain parts of Asia and is ubiquitously associated with the Epstein Barr virus (EBV). For recurrent and metastatic NPC, the platinum-based chemotherapeutic regimen is the mainstay of treatment. However, the median overall survival ranges from 15 to 19.6 months and most patients progressed despite chemotherapy with a median time to progression between 6 to 10 months in phase II clinical studies^1^. Therefore, better systemic therapy is needed.

Under the normal physiological condition, the Retinoblastoma gene (RB) controls the transit G1 checkpoint, which is subsequently phosphorylated by the cyclin-dependent kinase Cdk4/cyclin D and Cdk2/cyclin E complexes to release E2F and allow the expression of S-phase gene set^2^. Defects in the cell cycle are common in most solid cancers, and these may involve activation of Cdk4/6 or their regulatory D-type cyclins, loss of function of Cdk inhibitors like p16, p27, p53 loss, or excessive activity of Cdk4/6^3^. In NPC, whole exome sequencing (WES) studies have consistently shown that alterations of genes that regulate the cell cycle are also common in NPC^4,5^. In particular, the high prevalence of CCND1 amplification and cyclin D1 overexpression supports the role of CCND1 as an oncogene in NPC^6–8^. Deregulation of the cyclin-dependent kinase (Cdk) family of G_1_ checkpoint regulators has also been investigated in NPC. Zhang *et al*. reported that the G_1_/S phase regulators p16 and p27 are downregulated, while Cdk4 and RB genes are differentially upregulated in NPC tissues compared to the benign nasopharyngeal epithelium. At the protein level, RB, cyclin D1 and its regulator Cdk4 are overexpressed in 90.6%, 92% and 71%, respectively in NPC tissues^6^. Given the high cyclin D1 expression level, p16 inactivation and the wild-type status of p53 in nearly all EBV-associated NPC, inhibition of Cdk activity may potentially restore normal cell cycle regulation^3^. Clinically, Cdk inhibitor has been evaluated in advanced NPC. Seliciclib is an inhibitor of Cdk 2, 7 and 9, which has been shown to induce tumor shrinkage as well as induce tumor apoptosis, reduced expression of Mcl-1, cyclin D1 and pRB in paired tumor biopsies obtained following around 8 days of treatment^3,9^. However, further development of this agent in cancer has not eventuated.

Ribociclib (Ribociclib, Kisqali®, LEE011, Novartis) is an oral inhibitor of Cdk4/6 kinases that has been recently approved by the US Food Drug Administration for the first-line treatment of hormone-receptor positive, HER2 negative metastatic breast cancer when used in combination with aromatase inhibitor, based on the result of the phase III MONALEESA-2 study^10^. In preclinical tumor models, ribociclib could induce complete dephosphorylation of RB and inhibits cell growth via G_1_ arrest^2^. It is active in cancers harboring aberrations that increase Cdk4/6 activity e.g. cyclin D1 translocation. Ribociclib also shows anti-tumor activity in pRB-positive cancers driven by activated oncogenes that are located upstream, such as activating mutations of KRAS and inactivation of PTEN^11^. In this study, we investigated the effect of a Cdk4/6 kinase inhibitor, ribociclib (Ribociclib, Kisqali®, Novartis) in preclinical models of NPC. We hypothesized that given the prevalence of Cdk dysregulation in NPC and the preclinical activity observed with seliciclib in a past report, ribociclib should have activity in suppressing NPC growth, apoptosis and inducing cell cycle arrest. Given our previous report on the preclinical activity of the alpha-specific PI3K inhibitor, alpelisib (Alpelisib, BYL719, Novartis) in NPC^12,13^, we explored if there is any synergistic effect on tumor growth when combining ribociclib with alpelisib.

## Results

### Basal activation of RB and cell cycle regulatory proteins and effect of ribociclib on cell growth in NPC cell lines

**Figure 1:**
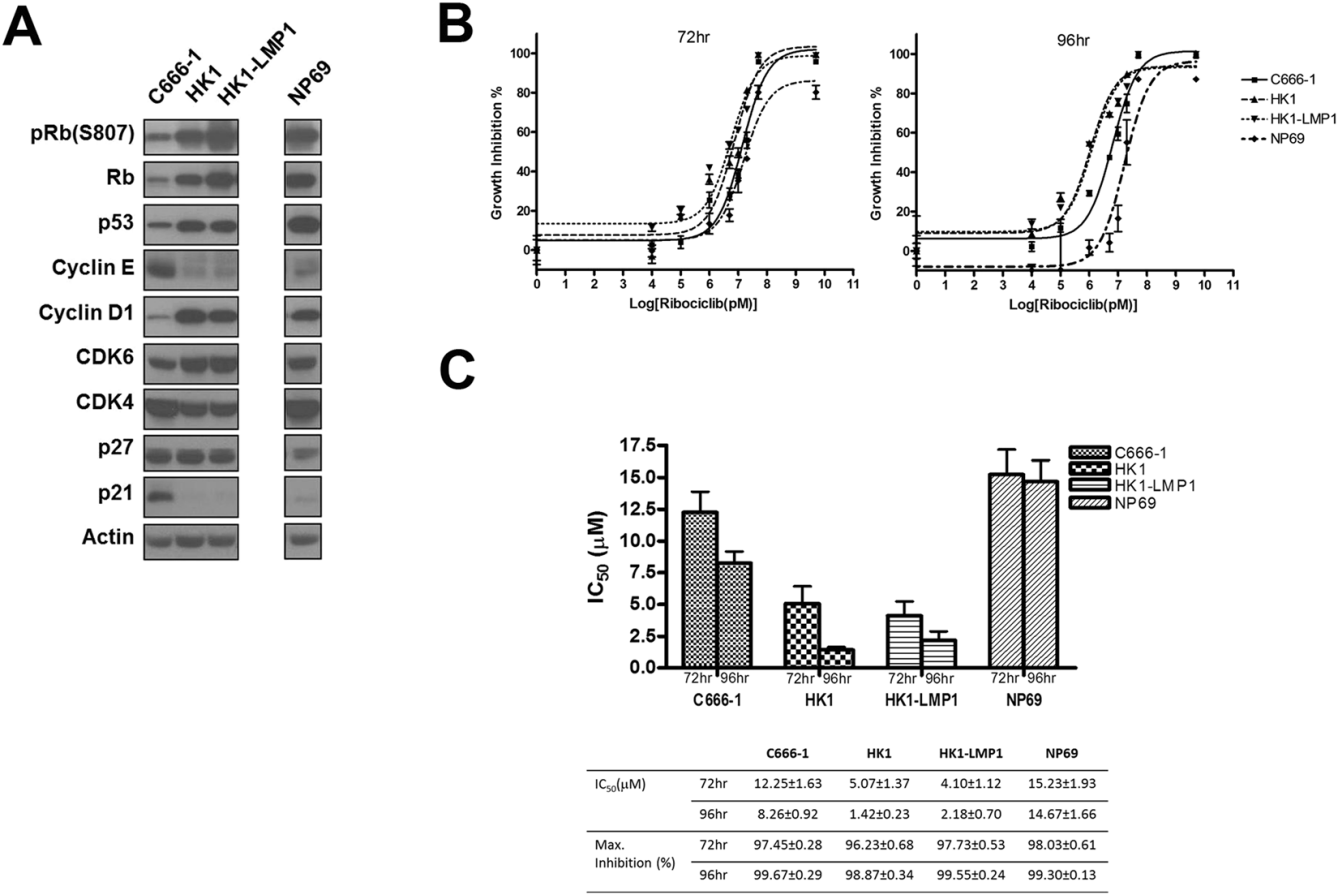
Cytotoxicity of a Cdk4/6 inhibitor, ribociclib, in NPC cell lines. (**A**) Basal cell cycle related protein expression of cancer cell lines C666-1, HK1, HK1-LMP1, and an immortalized epithelial nasopharyngeal epithelial cell line NP69 as normal control. pRb and cyclin D1 were expressed in all cell lines. (**B**) A representative dose-response curves showing the cytotoxicity effect of ribociclib in C666-1, HK1, HK1-LMP1, and NP69 by MTT assay. All samples were carried out in triplicate. (**C**) Corresponding IC_50_ and maximum growth inhibition for cell lines treated with ribociclib for 72 and 96 hours. Assays were run in three independent experiments and data are presented as mean ± SEM.

All cell lines used for *in vitro* test expressed major G_1_/S cell cycle proteins RB, cyclin D1, Cdk4, and Cdk6. Phosphorylation of RB (pRB) was detected in all cell lines, indicating their G_1_/S cell cycle transition was active. Tumor suppressor p53 weakly expressed in C666-1, while S phase Cdk2 regulators p21 and p27 were weakly expressed in all cell lines except the presence of p21 in C666-1 (Fig. 1A). Exposure to ribociclib for up to 72 hours resulted in over 95% of growth inhibition in all NPC cell lines. For treatment up to 96 hours, almost 100% of growth inhibition was observed in all cells (Fig. 1B). The respective IC_50_ values at 96 hours were in lower micro-molar range (in a descending order of sensitivity): HK1 = 1.42 ± 0.23 µM; HK1-LMP1 = 2.18 ± 0.70 µM; C666-1 = 8.26 ± 0.92 µM; NP69 = 14.67 ± 1.66 µM (Fig. 1B,C). HK1 was found to be the most sensitive to the growth inhibitory effect of ribociclib, and EBV positive C666-1 was also sensitive to Cdk4/6 inhibition. HK1 (most sensitive cell line with a high expression of pRB) and C666-1 (moderately sensitive with weaker pRB) were selected for further study of the effects of ribociclib in cell cycle signaling pathway.

### Ribociclib induced G_0_/G_1_ cycle arrest in NPC cell lines

**Figure 2:**
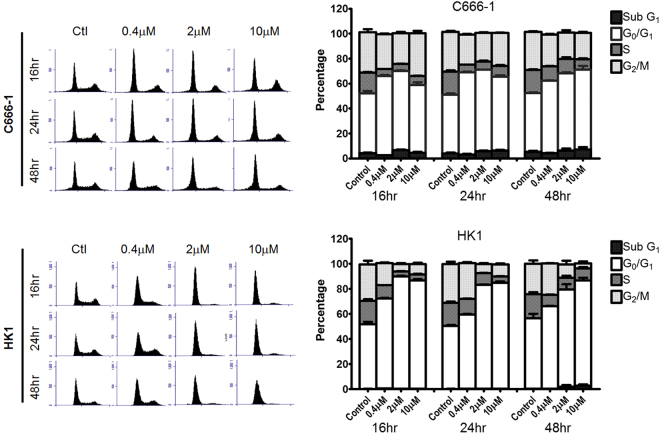
*In vitro* effects of ribociclib on cell cycle arrest. Representative graphs showing cell cycle distribution on C666-1 and HK1 cells treated with DMSO control and three different concentrations of ribociclib, one of them near their corresponding IC_50_, for 16, 24 and 48 hours. Cell cycle data are summarized from three independent trials and presented as mean ± SEM.

A time-dependent increase in the percentage of cells undergoing G_0_/G_1_ cycle arrest was observed in C666-1 in which the G_0_/G_1_ population had increased from 50% to more than 70% at 2 µM treatment but a higher dose at 10 µM did not effectively increase the G_0_/G_1_ cycle arrest. For HK1, ribociclib had more prominent effects in G_0_/G_1_ cycle arrest for all the time points analyzed, in which the G_0_/G_1_ population had increased from 50% to over 80% at both 2 µM and 10 µM treatment. (Fig. 2).

### Effects of ribociclib on cell cycle proteins

**Figure 3:**
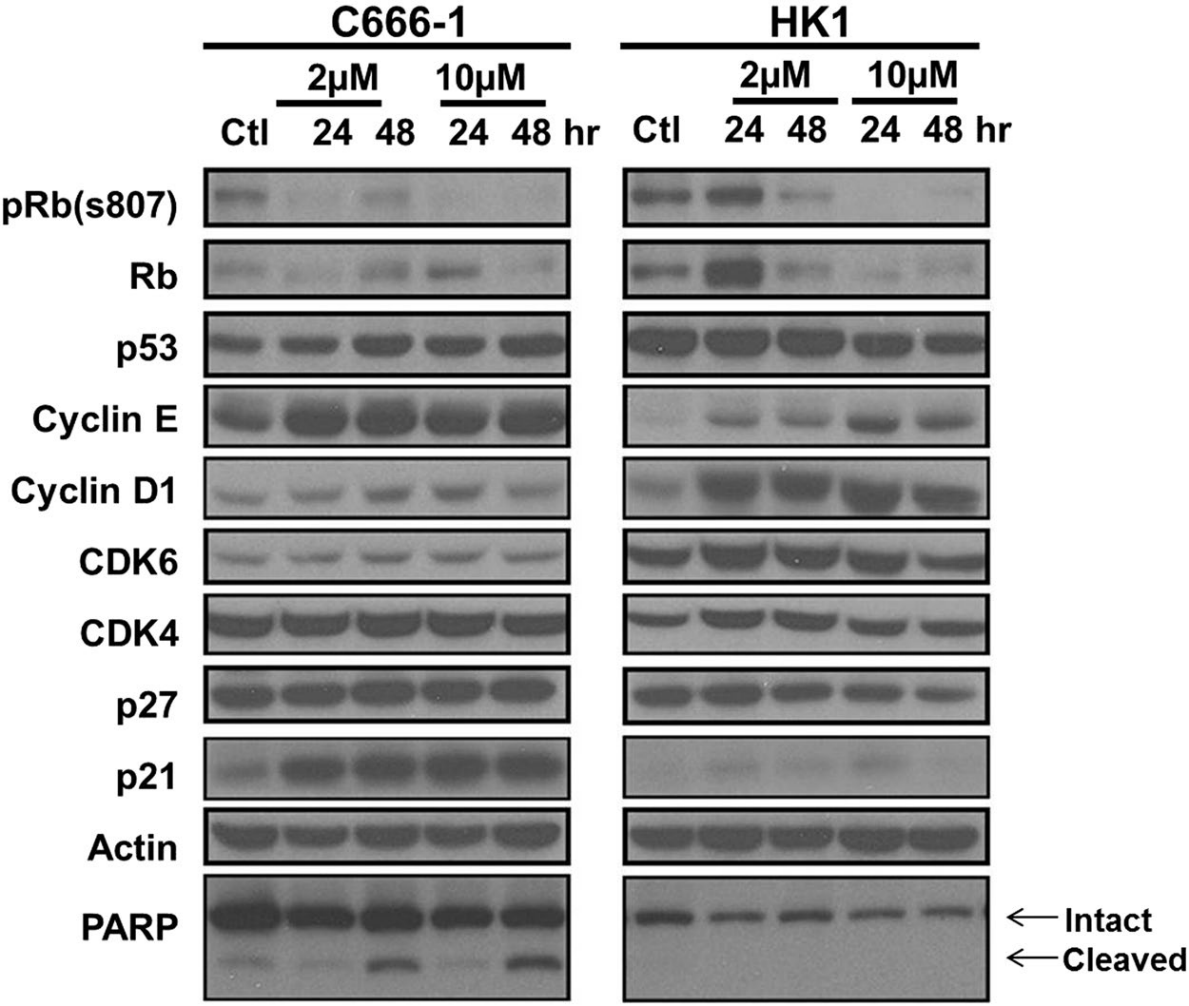
Effect of ribociclib on pRB and G_1_ cell cycle regulating protein expressions in C666-1 and HK1 cells. Cells were treated with ribociclib in 2 concentrations, one of them near their corresponding IC_50_, for 24 and 48 hours. Phosphorylation of Rb in all cell lines was significantly reduced by ribociclib but cyclin D1 and cyclin E expressions were upregulated. Cleaved PARP was moderately detected after 48 hours treatment in C666-1 but not in HK1.

The effect of ribociclib on G_1_/S cell cycle effectors and regulators was assessed by analyzing the expression level of cell cycle proteins. C666-1 and HK1 were treated with ribociclib at 2 µM and 10 µM for 24 and 48 hours. Upon treatment with ribociclib, the levels of phospho-RB were distinctly reduced to almost undetectable in C666-1 in both dosages and time points. For HK1, a longer incubation time and higher dosage will also inhibit RB phosphorylation effectively. The levels of total RB were significantly reduced except for a minor restore of RB levels in C666-1 at 24 hours with a higher dose treatment. An obvious increase in cyclin D1 was observed in HK1 after ribociclib treatment, but this is not a prominent observation in C666-1. On the other hand, minor increases in cyclin E levels were detected in all ribociclib treated samples. The levels of Cdk4 and Cdk6 remained similar in C666-1, but their levels were slightly increased in treated HK1. Ribociclib had minor effects in increasing the levels of cell cycle regulators such as p21 and p27. Ribociclib treatment had minimal effects on p53 expression level in C666-1 and HK1. Using cleaved PARP as an indicator for apoptosis, it was found that apoptosis was detected in C666-1 at 48 hours following exposure to ribociclib at both concentrations (Fig. 3).

### Inhibition of *in vivo* tumor growth in patient-derived xenografts using combined ribociclib and alpelisib treatment

**Figure 4:**
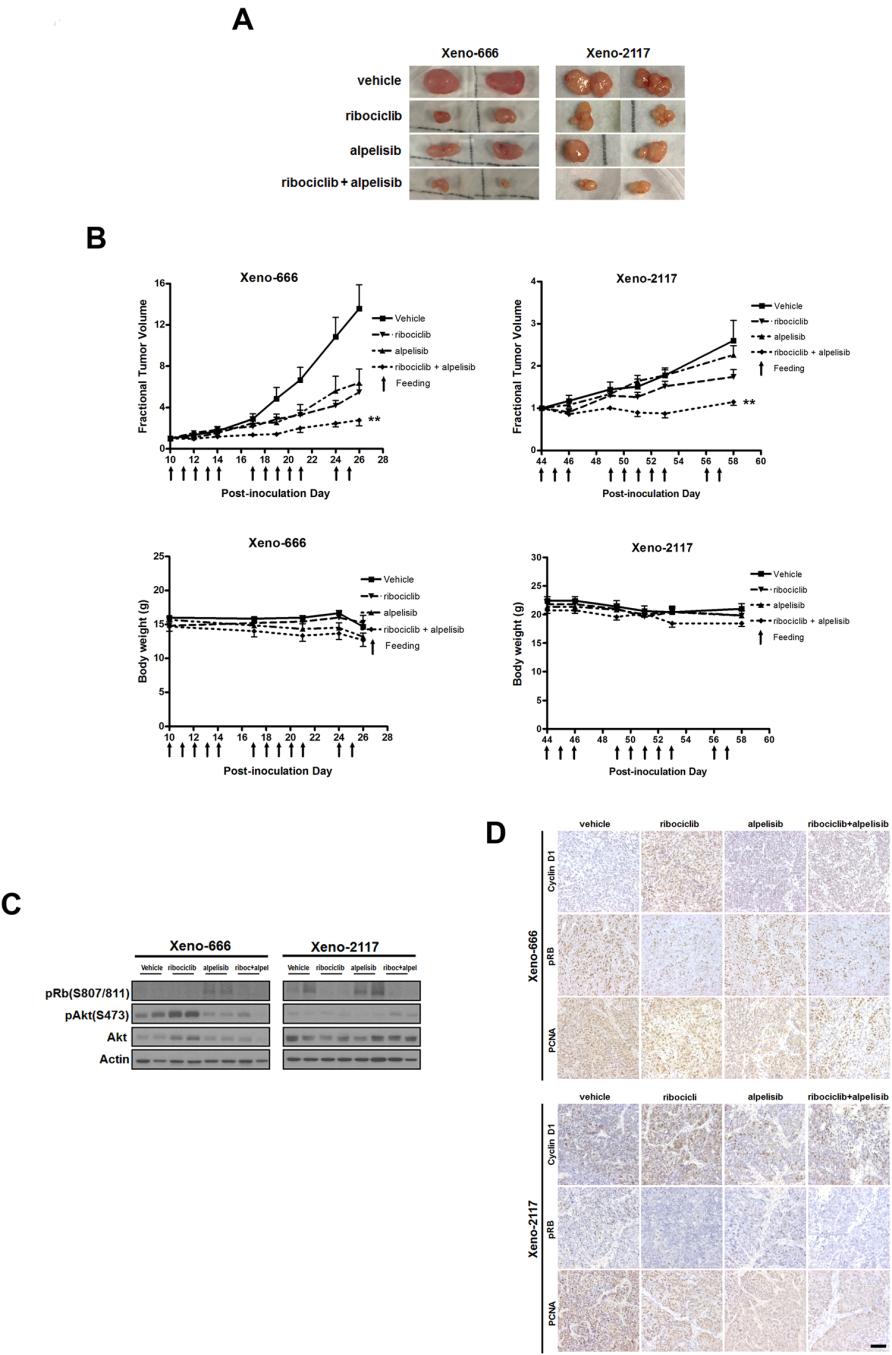
Combination of ribociclib and alpelisib inhibit the growth of NPC patient-derived xenografts. (**A**) Representative PDX tumors at the end point of drug treatment. Tumor volumes in both xeno-666 and xeno-2117 were significantly reduced by the combination treatment. (**B**) Mice were administered with vehicle, ribociclib, alpelsilib and combination treatment by oral gavage. Combination treatment can significantly reduce tumor volume in xeno-666 (**p < 0.01, ribociclib vs combination at post-inoculation day 26) and xeno-2117 (**p < 0.01, ribociclib vs combined at post-inoculation day 58). (Fractional tumor volume = tumor volume/tumor volume before drug treatment) Values were presented as mean ± SEM (n = 10 to 12 per group for xeno-666, n = 7 to 11 per group for xeno-2117 at end point, note that some mice were euthanized when tumors exceeded maximum allowable size considering animal ethics) The body weights of mice between groups were comparable in both PDX models, indicating the drug combination was well-tolerated. (**C**) Western blots for the xenografts after drug treatments. Significant reduction of pRb expression was observed in ribociclib groups. Alpelisib co-treatment had significantly reduced the ribociclib induced pAKT level. (**D**) Representative immunohistochemical staining images from PDX tissue. Significant reduction of pRb expression but the obvious increase in cyclin D1 was observed in both PDX tumors after ribociclib treatment. PCNA staining was significantly reduced in co-treated tumors, suggesting a reduction of cancer cell proliferation. (scale bar = 100 µm).

Two patient-derived xenograft models xeno-666 and xeno-2117 were used in testing the tumor growth inhibitory effects of ribociclib and alpelisib. The PDXs were provided by Prof. KW Lo’s laboratory, Department of Anatomical and Cellular Pathology, The Chinese University of Hong Kong. They were derived by undifferentiated NPC tumor biopsies from southern Chinese patients^14^. For xeno-666, drug administration started at 10 days after inoculation where the average tumor sizes were 30–40 mm^3^. Mice were divided into vehicle control group, ribociclib group (200 mg/kg), alpelisib group (50 mg/kg) and combination group. Mice in vehicle group were sacrificed at 26 days post inoculation due to excessive tumor growth, while a 2-week dosing schedule was completed for other treatment groups. For xeno-2117, drug administration started at 44 days post inoculation. Due to the variation of tumor sizes, mice bearing tumor with volume exceeding 1000 mm^3^ had been euthanized before the treatment began. Mice were sacrificed at 58 days post inoculation as the diameter of some tumors in vehicle group exceeded 15 mm. Mean tumor volumes of xeno-666 in vehicle control, ribociclib, alpelisib, co-treatment were 547 ± 134.1 mm^3^, 199 ± 39.6 mm^3^, 242.5 ± 57.5 mm^3^ and 108.2 ± 40.0 mm^3^ respectively. Mean tumor volumes of xeno-2117 in vehicle control, ribociclib, alpelisib, co-treatment were 815.4 ± 176.8 mm^3^, 451.7 ± 117.2 mm^3^, 856.7 ± 164.7 mm^3^ and 409.1 ± 79.7 mm^3^ respectively (data are presented as mean ± s.e.m., see Supplementary Fig. S2). Tumor measurement showed that either ribociclib or alpelisib could strongly inhibit the growth of xeno-666 tumors. Ribociclib alone is effective in xeno-2117 growth inhibition, but alpelisib did not show any prominent effects in this PDX tumor. Ribociclib and alpelisib co-treatment has shown a significant reduction in tumor volume compared with single treatment, with p = 0.007 (ribociclib vs combined) in xeno-666 and p = 0.0093 (ribociclib vs combined) in xeno-2117 (Fig. 4A,B). Both ribociclib and alpelisib single treatments as well as the combined treatment were well tolerated in the mice. The body weights in all treatment groups were comparable to vehicle treated group and remained unaffected relatively throughout the treatment, suggesting a minimal toxicity (Fig. 4B). The effects of *in vivo* drug treatments were further confirmed by Western blotting for tumor protein examinations. The levels of pRB were suppressed in ribociclib treated tumors and there was an obvious increase in cyclin D1 in both PDX tumors after ribociclib treatment. The addition of alpelisib did not antagonize the action of ribociclib in reducing the phosphorylation of RB, but successfully suppressed the Akt signaling where its phosphorylation was abolished, which contributed to the synergistic effects in PDX tumor growth inhibitions (Fig. 4C). The expressions of cyclin D1 and pRB were further confirmed by IHC staining of the xenograft tumor sections. PCNA staining was significantly reduced in co-treated tumors, suggesting a reduction of cancer cell proliferation (Fig. 4D).

## Discussion

In this study, ribociclib was effective in suppressing the growth of NPC cell lines at the lower end of micro-molar concentrations by inducing G_1_ cell cycle arrest and could significantly suppress RB phosphorylation. However, when the NPC cells were treated with ribociclib, we observed an accumulation of cyclin D1 over time, which may be due to an impaired cyclin D1 degradation process in G_1_ phase arrest. The accumulation of cyclin D1 would drive the cells into S phase once the inhibition of Cdk4/6 was diminished. Activation of the PI3K-Akt pathway is commonly observed in NPC patients, therefore, we hypothesized that the combination of ribociclib and the alpha-isoform specific PI3K inhibitor alpelisib may be synergistic. From our previous study, alpelisib was effective in suppressing cell growth in NPC cell lines at micro-molar concentrations, with around 80% of growth inhibition was attained at 72 hours of treatment for both 2D and 3D spheroids. It could induce apoptosis and G_0_/G_1_ cycle arrest in a dose dependent manner and effective in inhibiting the activation of Akt and its downstream pathways. The PI3K pathway has always been seen as a critical component in cell growth, proliferation, survival and protein synthesis^13^. Previous findings affirmed that Akt signaling and its downstream effectors are over-active in most NPC cell lines. In the current study, synergisms between ribociclib and alpelisib was observed in the PDX models of NPC. The additional suppression of Akt pathways by alpelisib had further strengthened the inhibitory effect. The rationale of combining Cdk4/6 inhibitors with an inhibitor that targets PI3K/Akt/mTOR axis is evidently working in preclinical studies. The combination of palbociclib with the TORC1 inhibitor everolimus synergistically inhibited NSCLC cell growth^15^. Ribociclib also demonstrated synergistic activity in PI3K inhibitor-resistant, PIK3CA-mutant breast cancer cell lines, with the PI3K inhibitors pictilisib (GDC-0941) or alpelisib. Tumor regression in PIK3CA-mutant breast cancer mouse models was observed^16^. Further investigations are necessary to evaluate the potential role of Cdk4/6 and PI3K pathway inhibitor combinations for treating breast cancer, at the same time, we are reporting the first use of Cdk4/6 and PI3K inhibitors in NPC model. Activation of the PI3K-Akt pathway has been proposed as a potential mechanism of resistance to Cdk inhibitors, therefore, the synergistic effects of ribociclib and the PI3K inhibitor alpelisib co-treatment in NPC PDX model was examined. Xeno-2117 is not very responsive to alpelisib alone compared to xeno-666. This is due to the absence of pAkt(S473) in xeno-2117, which is the major downstream regulator of the PI3K-Akt-mTOR cascade. The intrinsic low level of Akt phosphorylation suggested that xeno-2117 was quite independent of the Akt activation and therefore as a PI3K inhibitor, alpelisib was ineffective in tumor growth inhibition. However, the G_1_/S cell cycle regulators e.g. cyclin D1 and Cdk4/6 were highly expressed in xeno-2117, suggesting it is a good xenograft model to illustrate the effect of ribociclib. The combined treatment had a better growth inhibitory effect in xeno-666. From Fig. 4C, an induction of phospho-Akt level was observed after ribociclib treatment, which could enhance the cancer cell proliferation and render the growth inhibitory effect. Together with the lack of Cdk6 and weakly expressed cyclin D1, the effect of ribociclib alone was limited. In combination with alpelisib, the Akt activation was successfully suppressed, which contributed to an overall additive therapeutic effect.

Cyclin D1 controls cell cycle at the G_1_/S phase transition by interacting with Cdk4 and Cdk6 to phosphorylate RB, this results in the release of transcription factor E2F that is important for driving the cell in to express S phase gene sets to facilitate DNA replication. It also has some Cdk-independent functions including the regulation of various transcription factors, histone acetylases and deacetylases. In NPC, cyclin D1 and p16 have been identified as a target oncogene and a tumor suppressor gene respectively^17–19^. Hui *et al*. found that overexpression of cyclin D1 and amplification of its encoding gene CCND1 can be found in NPC cell lines, xenografts and over 90% of tumor tissues. Cyclin D1 is also overexpressed in premalignant, dysplastic nasopharyngeal epithelial cells (NPE) and may play an important role in the early pathogenesis of NPC. Moreover, cyclin D1 overexpression has been shown to suppress EBV-induced growth arrests and cellular senescence, and support stable EBV-infection in immortalized NPE cells^20^. The mechanism of cyclin D1 deregulation in NPC has been attributed to the activity of the EBV oncogenic protein, LMP-1, which has been shown to upregulate cyclin D1 transcription through the NFκB pathway and also increase Cdk4 expression *in vitro*^21–23^. On the other hand, p16 is frequently inactivated in NPC via CpG promoter methylation of the p16 gene^17,18^. The loss of p16 and cyclin D1 expression has been associated with increased risk of local recurrence in NPC following radiotherapy^24^. Despite this, older studies have found that the level of phosphorylated RB remains high in head and neck cancer^25^, with over 90% of tumors expressing pRB in one study^24^. RB controls whether a cell will transit through this checkpoint or not and during the G_1_ phase, RB is hypophosphorylated by the Cdk4/cyclin D complex. The Cdk2/cyclin complex then phosphorylates RB, which eventually drives the cell into S phase. Preclinical studies have suggested that RB overexpression, cyclin D1 overexpression, CCND1 amplification may be associated with increased susceptibility to the inhibitory effect of Cdk inhibitors. Therefore, regulators of the G_1_/M checkpoints may influence response to a Cdk4/6 inhibitor. Cdk inhibitors are currently entered phase III clinical development in chronic lymphocytic leukemia and possibly estrogen-receptor positive breast cancer^26^. An earlier generation of Cdk inhibitors such as flavopiridol or seliciclib was associated with bone marrow suppression, nausea, vomiting, and diarrhea, which were often dose-limiting. Targeting of Cdk4/6 may theoretically result in less normal tissue toxicity, as these Cdks are not essential for the cell cycle in normal cells. The proposed mechanism of action of Cdk4/6 inhibitors is to inhibit RB phosphorylation in the early G_1_ phase, thereby arresting the cell cycle in this phase^26^. Thus, Cdk4/6 inhibitors may behave predominantly as cytostatic rather than cytotoxic agents. It has been proposed that Cdk4/6 inhibitors are not expected to work in tumors lacking in RB and express a high level of wild-type p16. Cyclin D1 overexpression and CCDN1 amplification have been evaluated in the laboratory as biomarkers, but none of them has reliably predicted response to Cdk4/6 inhibitors. In small cell lung cancer cells, RB gene expression influences response to flavopiridol^27^. RB-negative cells tend to be most sensitive to flavopiridol-induced apoptosis. Certain subtypes of cancers such as ER positive, HER-2 positive breast cancer and KRAS induced lung cancer cells have been found to be particularly vulnerable to Cdk4/6 inhibition^26^. Given the high level of cyclin D1 overactivity, p16 inactivation and the wild-type status of p53 in nearly all EBV-associated NPC, inhibition of Cdk activity may potentially restore normal cell cycle regulation. In conclusion, the result of our study supports the clinical evaluation of ribociclib in combination with alpelisib in NPC.

## Methods

### Drugs, chemicals, and antibodies

Ribociclib and alpelisib were kindly provided by Novartis Pharma AG (Basal, Switzerland). RPMI-1640 medium, fetal bovine serum (FBS), sodium pyruvate, penicillin and streptomycin were from Thermo Fisher Scientific (Logan, Utah, USA). 3-(4,5-dimethylthiazol-2-yl)-2,5-diphenyltetrazolium bromide (MTT) was from Amersco (Solon, OH). Amersham ECL Western blotting detection reagents were purchased from GE Healthcare Biosciences (Pittsburgh, PA). The following antibodies were from Cell Signaling Technology (Danvers, MA): antibodies recognizing pRb(s780) (#9307), pRB(s807/811) (#9308), RB (#9309), cyclin D1(#2978), Cdk 4 (#12790), Cdk 6 (#3136), PARP (#9542), p21 (#2947), p27 (#3686), p-Akt(S473) (#4060), Akt(pan) (#4691), PCNA (#13110), β-tubulin (#2128). Cyclin E (#sc-274) and p53 (#sc-126) were bought from Santa Cruz Biotechnology while Actin (#CP01) was purchased from Calbiochem.

### Drug preparation

Ribociclib and alpelisib were dissolved in DMSO at concentrations of 50 mM and 20 mM respectively and stored in aliquot at −80 °C as recommended by the manufacturer. Aliquots were diluted in the corresponding medium just before addition to cell cultures.

### Cell lines

Two EBV-associated NPC cell lines (C666-1, HK1-LMP1), one non-EBV NPC cell line (HK1), and an immortalized epithelial nasopharyngeal epithelial cell line (NP69) were used in this study. The EBV genome in C666-1 cell line has been sequenced^28^.

### Cytotoxicity Assay

Cytotoxicity was assessed by MTT assay. Briefly, cells were cultured in 48-well plates (500–7000 cells per well) in the respective culture medium. Ribociclib in complete medium was added at 24 hours after cell plating and incubated at 37 °C with 5% CO_2_ for 72 and 96 hours. All samples were run in triplicate. Cell growth inhibition was expressed as the percentage of absorbance of control cultures measured at 570 nm with a microplate reader and 50% of the maximum growth inhibition (IC_50_) was calculated (GraphPad PRISM; GraphPad Software, Inc., La Jolla, CA).

### Cell cycle analysis

Cells treated with ribociclib were fixed with 70% cold ethanol and stained with propidium iodide (0.05 mg/ml) solution containing RNase (0.02 mg/ml). The analysis was performed using a Becton Dickinson Accuri C6 flow cytometer while data of cell cycle were processed and analyzed with their software.

### Immunoblotting

Total protein was collected by centrifugation of cells or tissue lysed with ice cold RIPA buffer. The lysate was then quantitated with bicinchoninic acid (BCA) assay Twenty-five microgram of total protein was resolved on an SDS-PAGE gel and transferred onto the Trans-Blot nitrocellulose membrane using wet transfer machine (BioRad Laboratories, Hercules, CA). The membrane was then blocked with non-fat dry milk, following overnight incubation of primary antibody at 4 °C. After rinsing, the membrane was then incubated with corresponding secondary antibody. The blot was developed with GE Amersham ECL chemiluminescent substrate by autoradiography.

### *In vivo* study of tumor xenograft

Five to seven weeks old female athymic nude mice (nu/nu) weighing about 16–20 grams were housed by The Laboratory Animal Services Centre of The Chinese University of Hong Kong. The experiment was conducted by researchers under license from the Hong Kong Government Department of Health and according to approval given by Animal Experimentation Ethics Committee of The Chinese University of Hong Kong. Briefly, about 3 mm diameter PDX xenograft was cut and minced into small pieces and then mixed with Hanks balanced buffer. The mixture was then inoculated into both flanks of nude mice. When tumor reached to suitable sizes, they were randomized into different groups for the treatment. The drug was prepared by suspending in 0.5% methylcellulose solution before administration. Either drug or vehicle was administered to the mouse by oral gavage on a schedule of 5 days on 2 days off for two weeks. Drug efficacy was evaluated by the method previously described^29^.

### Immunostaining of tumor tissue

Formalin fixed paraffin-embedded samples were de-paraffinized and rehydrated with a descending pecentages of ethanol from 100% to 0%. Following antigen retrieval and elimination of endogenous peroxidase, slides were blocked with BSA and incubated with an optimized dilution of primary antibodies at 4 °C overnight. The next day, samples were rinsed with PBST and incubated with DAKO REAL Envision HRP antibodies for 30 mins. The stain was visualized in brown with 3,3-diaminobenzidine (DAB) as substrate following counterstained with Mayer’s hematoxylin. After mounting, images were captured under the microscope Axio Observer Z1 (Carl Zeiss, Germany).

### Statistical analysis

Statistical analyses were performed using PRISM4 Software (GraphPad, La Jolla, CA). Unpaired T-test with Welch Correction was used unless specified. Findings were considered as statistically significant when P value < 0.05.

## Electronic supplementary material

supplementary info
