# Supplementary material for: Preclinical evaluation of ribociclib and its synergistic effect in combination with alpelisib in non-keratinizing nasopharyngeal carcinoma
Source: Sci Rep. 2018 May 22;8:8010. doi: 10.1038/s41598-018-26201-1 (PMC5964240; doi:10.1038/s41598-018-26201-1)

**Supplementary Information**

**Preclinical evaluation of ribociclib and its synergistic effect in combination with alpelisib in non-keratinizing nasopharyngeal carcinoma**

**Chi-Hang Wong ^1*,2^, Brigette B.Y. Ma ^1,2^, Connie W.C. Hui ^1,2^, Kwok-Wai Lo ^3^, Edwin P. Hui ^2^, Anthony T.C. Chan ^1,2^**

^1^ Cancer Drug Testing Unit (CDTU), Hong Kong Cancer Institute and Li Ka Shing Institute of Health Sciences, The Chinese University of Hong Kong, Hong Kong SAR, People’s Republic of China

^2^ Department of Clinical Oncology, State Key Laboratory in Oncology in South China, Sir YK Pao Centre for Cancer, The Chinese University of Hong Kong, Hong Kong SAR, People’s Republic of China

^3^ Department of Anatomical and Cellular Pathology, The Chinese University of Hong Kong, Hong Kong SAR, People’s Republic of China

Chi-Hang Wong and Brigette B.Y. Ma contributed equally to this work.

*Address correspondence to:

Chi-Hang Wong

Department of Clinical Oncology,

Prince of Wales Hospital,

Shatin, New Territories, Hong Kong SAR, China.

Tel: (852) 3505-1118; Fax: (852) 2648-8842

E-mail: [eric@clo.cuhk.edu.hk](mailto:eric@clo.cuhk.edu.hk)

Supplementary Fig. S1. Original basal CDK 4/6 pathway protein expressions in NPC/ NP cell lines.


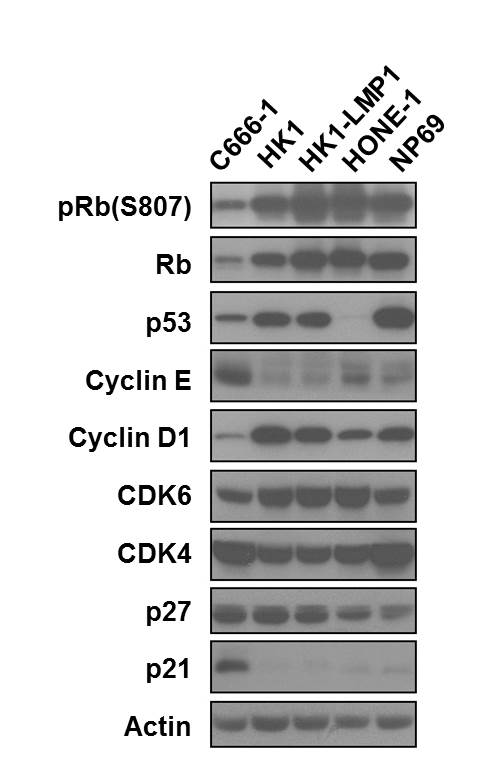


Supplementary Fig. S2. Co-treatment of ribociclib and aleplisib suppressed the tumor growth in two NPC PDX lines. The following charts showed the change in tumor volume after tumor implantation. Data are presented as mean ± sem.


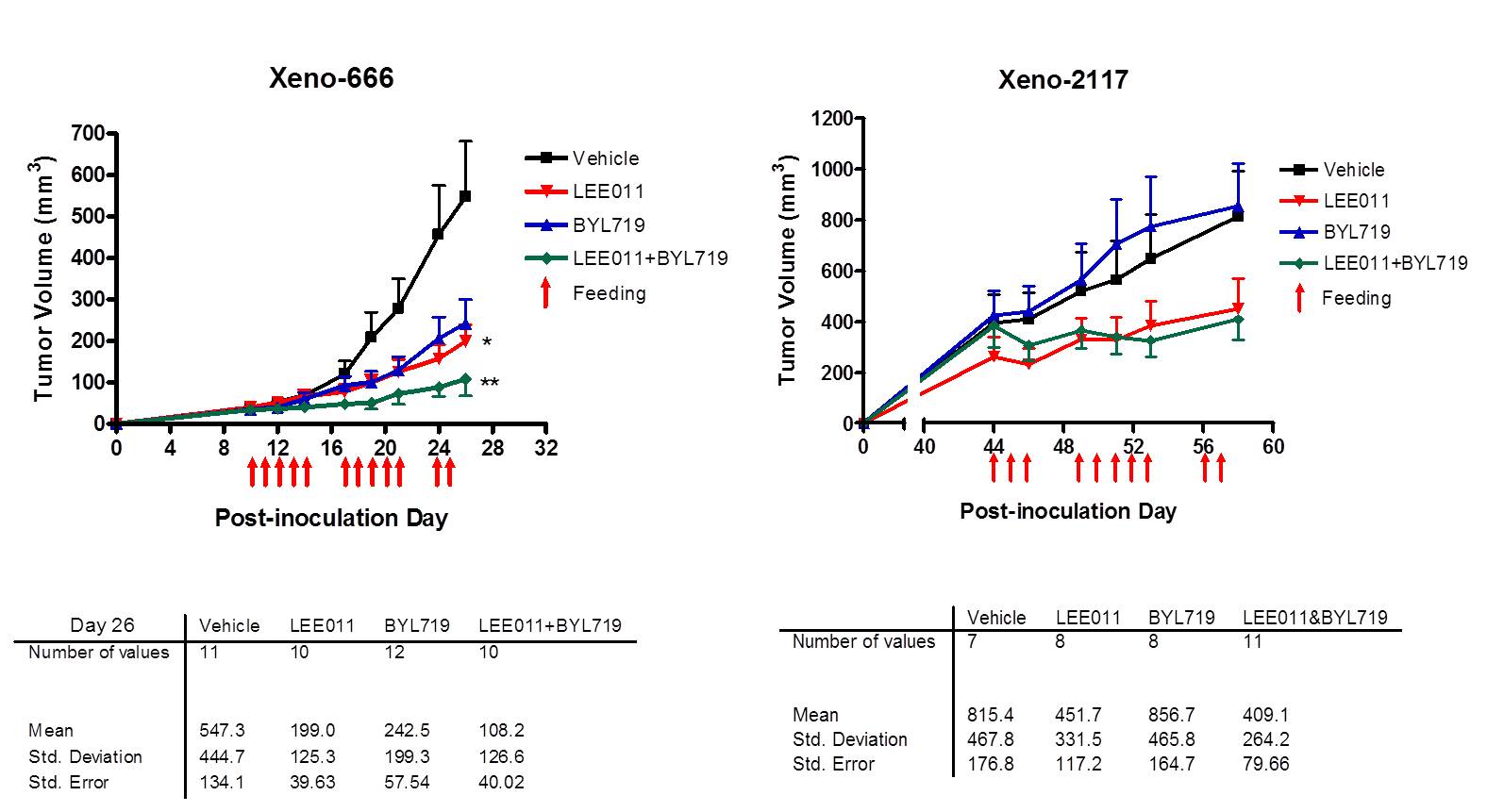

Supplement: Supplementary file 1 — supplementary info [file 41598_2018_26201_MOESM1_ESM.docx]
